# Determination of the Five Main Terpenoids in Different Tissues of *Wolfiporia cocos*

**DOI:** 10.3390/molecules23081839

**Published:** 2018-07-24

**Authors:** Ming Fu, Li Wang, Xianyou Wang, Boxia Deng, Xing Hu, Juan Zou

**Affiliations:** 1 College of Biological and Food Engineering, Huaihua University, Huaihua 418000, China; fm6988@163.com (M.F.); wangli4104@163.com (L.W.); dbx25802580@163.com (B.D.); 2 Key Laboratory of Research and Utilization of Ethnomedicinal Plant Resources of Hunan Province, Huaihua 418000, China; 3 Xiang-Gui-Qian Institute of Research in Edible and Pharmaceutical Fungi, Jingzhou 418400, China; wcocos@126.com

**Keywords:** *Wolfiporia cocos*, triterpenoids, pollution-controlled cultivation, sclerotium, fruiting body

## Abstract

*Wolfiporia cocos* is a fungus containing triterpenoids and is widely used as an herbal medicine. However, it is unknown whether its main triterpenoid contents differ in different tissues. In this study, we identified dehydrotumulosic acid, polyporenic acid C, pachymic acid, dehydrotrametenolic acid, and dehydroeburicoic acid as the five main triterpenoids in *W. cocos*. We also systematically profiled the contents and distribution of these main triterpenoids in different tissues of *W. cocos*. High contents of all five triterpenoids were found in the surface layer of *W. cocos*. Intriguingly, we noted that the highest contents of the five triterpenoids were found in the surface layer of the sclerotium grown under pollution-controlled cultivation; the second-highest contents were found in the surface layer of the natural sclerotium. These results indicate that environmentally friendly cultivation of the sclerotium of *W. cocos* is a practical way to increase the productivity of *W. cocos*. In addition, our findings suggest that the triterpenoids may contribute to the pharmacological activity of *W. cocos*, and the surface layer of sclerotium in *W. cocos* might be a promising raw material for applications in health care and the development of functional medical products.

## 1. Introduction

*Wolfiporia cocos* (F.A. Wolf) Ryvarden & Gilb is an edible and medicinal mushroom growing on the roots of pine trees and has been widely used as an herbal medicine in China, Japan, Korea, and North America [1]. It is enriched with various organic compounds and is used in many traditional Chinese prescriptions to treat gastritis, nephrosis, edema, dizziness, nausea, emesis and hyperglycemia in many traditional Chinese prescriptions [2,3,4,5,6]. According to the 2010 edition of the *Chinese Pharmacopoeia*, more than 10% of traditional Chinese medicine reparations include *W. cocos* [7,8]. Triterpenoids in *W. cocos* are a class of natural compounds that contain acyclic 30-carbon precursors. Pachymic acid, a lanostane-type triterpenoid also found in *Fomitopsis pinicola*, is one of the important chemical functional components of *W. cocos* [9,10]. Ling et al. (2011) reported that pachymic acid inhibits breast cancer cell invasion by suppressing nuclear factor-κB-dependent matrix metalloproteinase-9 expression [11]; Hong et al. (2012) demonstrated that pachymic acid reduces breast cancer cell metastasis via suppression of the phosphorylation of PITPNM3 and the interaction of CCL18 with PITPNM3 [12]. These results suggest that *W. cocos* may have anti-cancer and anti-inflammatory properties. Besides pachymic acid, *W. cocos* also contains other triterpenoids such as dehydrotumulosic acid, polyporenic acid C, dehydrotrametenolic acid, and dehydroeburicoic acid. Dehydrotumulosic acid, an inhibitor of phospholipase A_2_, is a natural anti-inflammatory agent [13]. Polyporenic acid C was shown to significantly restrain the growth of human lung cancer A549 cells and induce caspase-8-mediated apoptosis through the death receptor-mediated apoptotic pathway [14]. Kang et al. (2006) reported that dehydrotrametenolic acid could selectively reduce H-ras-transformed cell proliferation through cell cycle arrest at the G2/M phase and accumulation of sub-G1 population by suppressing H-ras signaling pathways [15]. Deng et al. (2009) found that dehydroeburicoic acid inhibits the proliferation of human glioblastoma U87MG cells and induces necrotic cell death, which involves Ca^2+^ overload, mitochondrial dysfunction, and calpain activation [16]. These studies of the active compounds in medical herb plants have triggered wide interest in exploring the contents and distribution of the important components, especially triterpenoids, in different tissues of *W. cocos*.

Jingzhou County in Hunan province is currently the largest *W. cocos* planting area and processes 78,000 tons of *W. cocos* per year, accounting for 70% of China’s poria products [17]. However, the sclerotia of *W. cocos* are usually cultivated by inoculation of the roots of pine trees with poria fungus, which can cause serious damage to the environment and natural resources [18]. To meet the increasing demand for *W. cocos* and remain environmentally friendly while increasing productivity, the cultured mycelium and pollution-control cultivation of *W. cocos* sclerotia are needed. Pollution control is the process of reducing or eliminating the release of pollutants into the environment. In this study, pollution-controlled cultivation of *W. cocos* sclerotia refers to cultivation of the sclerotia using forestry wastes such as tree pockets, branches, and sawdust in local mountain slope areas without the application of herbicides, pesticides, plant growth regulators, or the destruction of pine trees or other forest species [17]. Moreover, due to the difficulty of culturing the fruiting body of *W. cocos*, the contents of triterpenoids in *W. cocos* have only been evaluated in the sclerotia of *W. cocos*, and the triterpene contents in the fruiting body among the different tissues remain to be determined. In the present study, we used the cultured mycelium and pollution-controlled cultivation of *W. cocos* sclerotia as possible substitutes for natural *W. cocos* production. We further determined the composition, contents, and distribution of terpenoids in different tissues of *W. cocos*, and assessed the triterpene contents in the fruiting body.

## 2. Results and Discussion

### 2.1. Qualitative and Quantitative Analysis

Using reference standards, the HPLC (wavelength = 241 nm) profiles of the five investigated triterpenoids were determined by linear range, regression equation, limit of detection (LOD), and limit of quantification (LOQ). The results showed that the correlation coefficients of the five investigated triterpenoids were above 0.999, indicating the good linearity of triterpenoids (Table 1). The LOD and LOQ ranges of the five triterpenoids were 0.1–1.375 µg/mL and 1–10 µg/mL (Table 1); the recovery rates of dehydrotumulosic acid, polyporenic acid C, pachymic acid, dehydrotrametenolic acid, and dehydroeburicoic acid were 111.6%, 92.5%, 102.2%, 103.9%, and 92.1%, respectively (Table 2).

**Figure 1:**
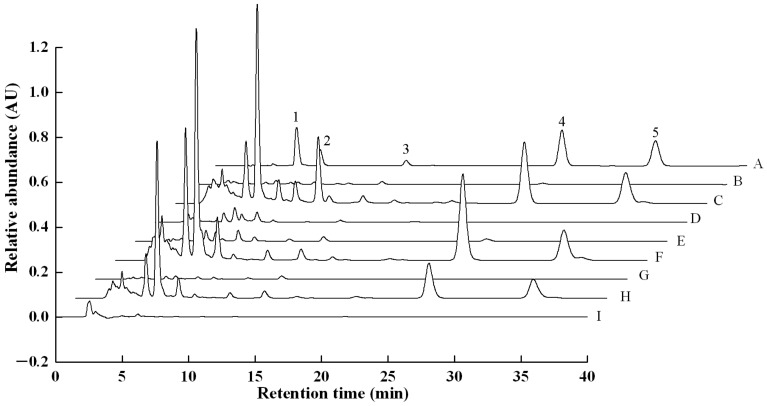
HPLC profiles of reference standards and the extraction of the terpenes of *W. cocos* (wavelength = 241 nm). 1, dehydrotumulosic acid; 2, polyporenic acid C; 3, pachymic acid; 4, dehydrotrametenolic acid; 5, dehydroeburicoic acid; A, standard samples; B, natural sclerotium; C, surface layer of natural sclerotium; D, cultured mycelium; E, pollution-control cultured sclerotium; F, surface layer of pollution-control cultured sclerotium; G, bionic cultured sclerotium; H, surface layer of bionic cultured sclerotium; I, matured fruiting body.

In Figure 1, in comparison to standard samples (A), natural sclerotium (B), cultured mycelium (D), pollution-control cultured sclerotium (E), bionic cultured sclerotium (G), matured fruiting body (I) showed small or rare peaks of terpenes compounds, while in surface layer of natural sclerotium (C), surface layer of pollution-control cultured sclerotium (F) and surface layer of bionic cultured sclerotium (H), peaks of five triterpenoids were all found, especially much higher relative absorbance of dehydrotumulosic acid was present. These results indicate that the activity of five investigated triterpenoids are apparently related to tissues of *W. cocos*.

### 2.2. Contents of Five Triterpenoids in Different Tissues

As shown in Figure 1, both natural sclerotium and pollution-controlled cultured sclerotium gad had a very high peak of relative abundance at retention time between 5 and 10 min. In pollution-controlled cultured sclerotium, the surface layer abundances of dehydrotumulosic acid, polyporenic acid C, pachymic acid, dehydrotrametenolic acid, and dehydroeburicoic acid were 14.8 ± 0.8 g/kg; 18.1 ± 0.6 g/kg; 17.6 ± 1.3 g/kg; 21.2 ± 1.5 g/kg, and 9.1 ± 0.7 g/kg, respectively. In natural sclerotium, the abundances of the five triterpenoids in the surface layer were 12.3 ± 0.3, 0.9, 1.3, 13.5 ± 0.8, and 8.5 ± 0.2 g/kg (Table 3).

The bionic cultured sclerotium, cultured mycelium and matured fruiting body displayed roughly similar amount of dehydrotumulosic acid with range from 0.193 to 0.217 g/kg. The contents of pachymic acid, polyporenic acid C, and dehydrotrametenolic acid in the surface layer of pollution-controlled cultured sclerotium were 17.6 ± 1.3 g/kg, 18.1 ± 0.6 g/kg and 21.2 ± 1.5 g/kg respectively, which were much higher than the contents of the others. The contents of dehydrotumulosic acid, polyporenic acid C, pachymic acid, and dehydrotrametenolic acid in the matured fruiting body were significantly lower than the contents in the sclerotia and surface layers of *W. cocos*. Polyporenic acid C and dehydrotrametenolic acid were not detected in the cultured mycelium of *W. cocos*. Dehydroeburicoic acid was only found in the three types of surface layers of sclerotia (Table 3).

### 2.3. Verification of the Five Triterpenoids

**Figure 2:**
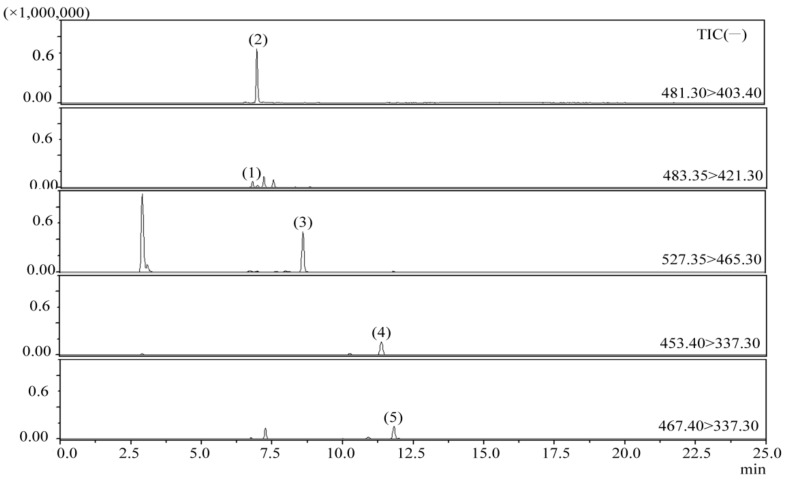
Chromatographic profile of representative samples from multiple reaction monitoring (MRM) method, ions extracted from full scan chromatogram: (1) dehydrotumulosic acid; (2) polyporenic acid C; (3) pachymic acid; (4) dehydrotrametenolic acid; (5) dehydroeburicoic acid.

To verify the five peaks in *W. cocos*, HPLC peaks were collected as eluate from the column outlet. Part of the solvent collected was removed by rotary evaporation, and then the concentrate was re-analyzed by ESI-MS/MS. Chromatographic profiles of representative samples from the multiple reaction monitoring (MRM) method are shown in Figure 2. Electrospray ionization-tandem spectroscopy ions of the five investigated triterpenoids in *W. cocos* are displayed in Table 4.

In comparison to the retention times and MS characteristics of the reference standards, peaks 1, 2, 3, 4, and 5 were identified as dehydrotumulosic acid, polyporenic acid C, pachymic acid, dehydrotrametenolic acid, and dehydroeburicoic acid (Table 4).

## 3. Materials and Methods

### 3.1. Materials

**Figure 3:**
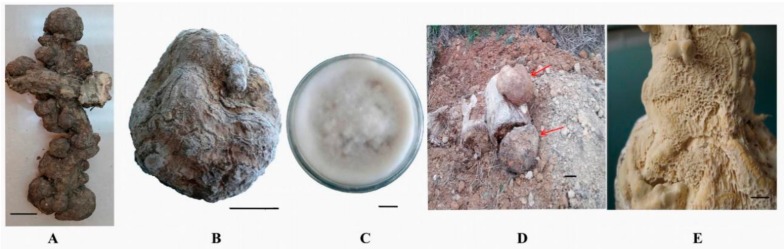
*Wolfiporia cocos*. (**A**) natural sclerotium of *Wolfiporia cocos*; (**B**) bionic cultured sclerotium; (**C**) colony on PAD medium; (**D**) pollution-control cultured sclerotium; (**E**) cultured fruiting body. Bars: A, B, D = 5 cm; C, E = 1 cm.

The natural fresh sclerotium (Figure 3A) and bionic cultured sclerotium (Figure 3B) of *W. cocos* were provided by the Xiang-Gui-Qian Institute of Research in Edible and Pharmaceutical Fung, Jingzhou, Hunan, China. The strain, named XPD007-2010 (GenBank number KX268225) (Figure 3C), was isolated from the natural sclerotium of *W. cocos*. The mycelium-bearing sclerotium of *W. cocos*, cultivated in artificial media, was collected (Figure 3D) as previously described [18].

### 3.2. Inoculum Preparation and Flask Cultures

The medium for agar plates contained (g/L) potato infusion, 200; glucose, 20; and agar, 20. The seed medium contained (g/L) potato infusion, 200; microcrystalline fiber, 5; glucose, 15; (NH_4_)_2_SO_4_, 1; MgSO_4_·7H_2_O, 1; and KH_2_PO_4_, 0.5 at pH 6.5. The production medium (g/L) was comprised of potato infusion, 200; microcrystalline fiber, 10; glycerol, 0.2; glucose, 30; (NH_4_)_2_SO_4_, 2; KH_2_PO_4_, 1; MgSO_4_·7H_2_O, 1.5; KNO_3_, 1; VB_1_, 0.006; VB_2_, 0.003; VB_6_, 0.0004; and nicotinamide, 0.02 at pH 6.5. The strain of XPD007-2010 was initially cultured in an agar plate at 28 °C for 5–6 days. The agar plate culture (5 mm) was added into the seed medium (50/250-mL flask) and incubated at 28 °C and 150 r/min for 10 days. The production medium in a 50/250-mL flask was inoculated with 7% (*v/v*) of the seed liquid and cultivated at 180 r/min and 28 °C for seven days. The fermented mycelium of *W. cocos* was obtained by centrifugation at 4000× *g* for 10 min.

### 3.3. Artificial Cultivation of Fruiting Body

The solid medium consisted of (g/bag): pine wood chips, 200; corn, 200; bran, 85; sucrose, 5; CaSO_4_·2H_2_O, 5; Ca(H_2_PO_4_)_2_·H_2_O, 3.5; and MgSO_4_·7H_2_O. A spawn bag (17 cm in width and 33 cm in length) was filled with 500 g of solid medium and 300 mL of water. The spawn bags were sterilized at 121 °C for 2 h. The sterilized spawn bag was inoculated with an agar block (5-mm-diameter) containing mycelial mats of *W. cocos* and cultivated for 30 d at 25 °C.

For fruiting body development, the solid medium covered by mycelium was cultivated with light intensity of 100–400 lx at 28 °C for 60 days. The fungus-bearing fruiting body of *W. cocos* was obtained from artificial media (Figure 3E).

### 3.4. Extraction of Triterpenoids of *W. cocos*

All samples, dehydrated to a stable weight, were mashed by hand with a pestle and mortar. Three grams of ground samples (natural sclerotium and the surface layer, bionic cultured sclerotium and the surface layer, pollution-controlled cultured sclerotium and the surface layer, cultured mycelium, and the fruiting body of *W. cocos*) were weighed accurately and soaked in 30 mL of methanol for 1 h. Following 30 min of ultrasonic treatment at 40 °C, the mixture was maintained at a constant weight by adding methanol and centrifuged at 4000× *g* for 15 min. The supernatant was filtered through a micropore filter (0.22 µm), then diluted 1 to 10 times with methanol and analyzed by HPLC.

### 3.5. HPLC Analysis

Standard samples of dehydrotumulosic acid, polyporenic acid C, pachymic acid, dehydrotrametenolic acid and dehydroeburicoic acid (Chengdu Desite Biotech Co., Ltd., Chengdu, China) were verified by reverse phase HPLC using a Waters Series HPLC (Waters, Milford, MA, USA) rigged with a Waters 1525 binary high-pressure pump and a Waters 2489 UV-Vis detector (Waters, Milford, MA, USA), and performance was operated by Breeze 2 software (Waters, Milford, MA, USA). Separation was performed on a C18 column (Diamonsil 250 mm × 4.6 mm, 5 µm; Dikma, Foothill Ranch, CA, USA). The absorbance of the eluate was determined at 241 nm [19,20]. The mobile phase and elution profile were described as follows: the mobile phase consisted of 0.1% formic acid–acetonitrile aqueous solution (20:80, *v*/*v*). The mobile phase was degassed by sonication before use. The column temperature was at 30 °C. The eluent flow rate was kept constant at 1.0 mL/min, and the volume of sample injection was 10 µL. Each sample was repeated three times to insure reproducibility.

### 3.6. Standard Samples and the Linear Regression Equation

**Figure 4:**
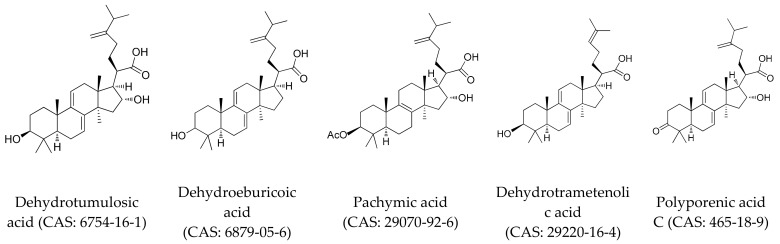
The structures of dehydrotumulosic acid, dehydroeburicoic acid, pachymic acid, dehydrotrametenolic acid, and polyporenic acid C.

Standard samples of dehydrotumulosic acid, polyporenic acid C, pachymic acid, dehydrotrametenolic acid, and dehydroeburicoic acid were dissolved in methanol/chloroform (10:1, *v*/*v*) solution. The chemical structures of the five reference standards are displayed in Figure 4. The recovery, the reproducibility and the linearity for the five investigated constituents were evaluated. The calibration curves (analyte to peak area ratio versus concentration) over the control range of the five triterpenoids were calculated. Triterpenoids were confirmed by comparing the retention time, purity coefficient, and spectrum with the known reference standards. The external standard method was employed for the quantification of the investigated constituents in *W. cocos*.

### 3.7. LC/MS-MS Analysis

For the HPLC analysis, a Shim-pack GIST C18 column (100 mm × 2.1 mm, 1.9 μm, Shimadzu, Kyoto, Japan) was adopted. The mobile-phase that contained methanol (A) and ultrapure water (B) was ultrasonically degassed prior to use. The samples were chromatographed under the following gradient conditions: 0–4 min, linear from 50% to 90% A; 4–8 min, 90% A; 8–11 min, linear from 90% to 100% A; 11–20 min 100% A; and 20–24 min, linear from 100% to 50% A; and 24–25 min, 50% A for equilibrating the column. The flow rate was 0.30 mL/min, the sample-tray temperature was maintained at 4 °C, and the sample volume was 1.0 μL.

MS was carried out on an LC-MS/MS8045 (Shimadzu, Kyoto, Japan) equipped with an ESI source manipulated in both positive-ion and negative-ion mode. The scan range for MS was varied from *m*/*z* 200 to *m*/*z* 650. Analysis was performed in PIS mode. The source parameters were shown below: DL temperature, 250 °C; heat block temperature, 400 °C; drying gas flow, 10.0 L/min; spray gas pressure, 35 psi; and capillary voltage, 4.0 kV.

## 4. Conclusions

At present, there are few reports on the contents of triterpenoids from *Wolfiporia cocos*. Most studies on triterpenoids mainly focus on their molecular structures and pharmacological activities. Li et al. (2015) reported that the contents of pachymic acid from the sclerotium of *W. cocos* from different areas were 0.198–0.332% (1.98–3.32 g/kg), significantly lower than in the epidermis (5.66–8.09 g/kg) [21]. Liu et al. (2015) found that the contents of pachymic acid in different grades of poria from different areas were 1.25–2.85 g/kg [22]; Xuet al. (2010) demonstrated that the contents of pachymic acid from 27 kinds of strains of *W. cocos* were 1.46–2.99 g/kg [19]. Li et al. (2015) reported that the pachymic acid content in white poria piece and epidermis were 0.79 g/kg and 10.21 g/kg, respectively [23]. In our experiment, we found that the pachymic acid content in the sclerotia (0.45–9.33 g/kg) was obviously lower than that in the epidermis (1.12–17.59 g/kg), which was consistent with the results obtained by Li, M. et al. (2015) and Li, X. et al. (2015). Furthermore, we also found that dehydrotumulosic acid, pachymic acid, dehydrotrametenolic acid, and dehydroeburicoic acid had low solubility (<5 µg/µL) in methanol, while these reference standards were well dissolved in the solution of methanol/chloroform (10:1, *v*/*v*), our calibrated reference standard may be an explanation for the inflated values of pachymic acid content reported in some earlier studies. The concentration of all five triterpenoids in the tested sample solution was lower than 2.5 µg/µL, which could be well dissolved in methanol, and recoveries for the five triterpenoids proved that in our experiment.

In this study, we identified dehydrotumulosic acid, polyporenic acid C, pachymic acid, dehydrotrametenolic acid, and dehydroeburicoic acid as the five main terpenoids in *Wolfiporia cocos*. Our results on the contents and distribution of the five terpenoids in different tissues suggest that *W. cocos* bearing the sclerotium or fruiting body might contain active terpenoids, and be used as a reference or be incorporated into alternatives for Chinese medicine. Moreover, the contents of the five triterpenoids in the surface layer of the sclerotium were much higher than in other parts of the sclerotium. Our findings indicate that the surface layer of the sclerotium in *W. cocos* is a promising raw material of *W. cocos* products and provides the basis to explore the pharmacological value of the triterpenoids in the surface layer of *W. cocos* in further study.

## Figures and Tables

**Table 1:** Linear range, regression equation, LOD, and LOQ for the five investigated triterpenoids by reverse phase HPLC-UV/Vis detector.

Analyte	Linear Regression Data			LOD (µg/mL)	LOQ (µg/mL)
	Regression Equation	r^2^ (*n* = 9)	Linear Range (µg/mL)		
Dehydrotumulosic acid	Y = 12608x + 25719	0.9992	4.575–305	0.305	1.525
Polyporenic acid C	Y = 22452x + 24889	0.9997	2–200	0.1	1
Pachymic acid	Y = 7488.6x − 10819	0.9996	10–800	3	10
Dehydrotrametenolic acid	Y = 7045.4x + 38355	0.9992	8.25–550	1.375	8.25
Dehydroeburicoic acid	Y = 6838.8x + 68109	0.9993	5.25–700	0.525	3.5

**Table 2:** Precision, repeatability, stability, and recovery of the five investigated analytes.

Analyte	Precision (RSD, %, *n* = 6)	Stability (RSD, %, *n* = 6)	Repeatability (RSD, %, *n* = 6)	Recovery (%, *n* = 3)	
	Intraday			Mean	RSD (%)
Dehydrotumulosic acid	2.32	2.69	4.72	111.6	4.73
Polyporenic acid C	2.40	4.59	2.20	92.5	2.06
Pachymic acid	2.96	3.20	2.52	102.2	5.40
Dehydrotrametenolic acid	1.93	3.07	2.98	103.9	4.68
Dehydroeburicoic acid	1.90	3.03	2.79	92.1	1.96

**Table 3:** Contents (mg/kg) of five triterpenoids in different tissues of *W. cocos.*

Analyte	Dehydro-Tumulosic Acid	Polyporenic Acid C	Pachymic Acid	Dehydro-Trametenolic Acid	Dehydro-Eburicoic Acid
Natural sclerotium	344.0 ± 22.0	93.2 ± 2.7	588.9 ± 41.5	352.7 ± 18.3	nd
Surface layer of natural sclerotium	12,300.7 ± 285.9	955.9 ± 21.0	1344 ± 27.6	13,534.4 ± 815.4	8514.6 ± 237.0
Bionic cultured sclerotium	193.0 ± 18.1	86.7 ± 7.3	445.9 ± 23.0	56.3 ± 3.4	nd
Surface layer of bionic cultured sclerotium	9388.8 ± 651.9	829.9 ± 56.2	1119.5 ± 104.6	8265.5 ± 591.8	5759.1 ± 411.3
Pollution-control cultured sclerotium	597.3 ± 45.0	428.0 ± 12.1	9332.5 ± 623.0	534.6 ± 48.7	nd
Surface layer of pollution-control cultured sclerotium	14,819.8 ± 833.8	18,120.0 ± 592.0	17,594.3 ± 1295.6	21,160.7 ± 1545.4	9060.9 ± 690.2
Cultured mycelium	216.6 ± 10.5	nd	203.7 ± 1.9	nd	nd
Matured fruiting body	216.6 ± 5.4	15.7 ± 2.0	197.6 ± 11.7	19.8 ± 1.6	nd

Means ± standard deviation (*n* = 3); nd, not detected.

**Table 4:** ESI-MS/MS data of constituents identified from the collected eluates of *W. cocos.*

Analyte	*t*_W_/*t*_R_ (min)	Formula	Ion Type	*m*/*z* (Experimental/Calculated)	Product Ion MS/MS (*m*/*z*)
Dehydrotumulosic acid	6.660/6.662	C_31_H_48_O_4_	[M − H]^−^	483.35/483.35	421.30, 423.40, 337.40
Polyporenic acid C	6.846/6.826	C_31_H_46_O_4_	[M − H]^−^	481.30/481.30	403.40, 387.35
Pachymic acid	8.374/8.399	C_33_H_52_O_5_	[M − H]^−^	527.35/527.35	465.30, 467.35
Dehydrotrametenolic acid	11.153/11.147	C_30_H_46_O_3_	[M − H]^−^	453.40/453.40	337.30, 371.25
Dehydroeburicoic acid	11.683/11.639	C_31_H_48_O_3_	[M − H]^−^	467.40/467.40	337.30, 339.30

*t*_R_ is the retention time of the standard samples; *t*_W_ is the retention time of analytes in *W. cocos.*
